# Construction of Regular Hexagonal Double-Layer Hollow Nanocages by Defect Orientation and Composite Phase Change Materials with Carbon Nanotubes for Thermal Safety of Power Batteries

**DOI:** 10.3390/nano16010026

**Published:** 2025-12-24

**Authors:** Silong Wang, Wei Yan, Pan Sun, Jun Yuan

**Affiliations:** School of Nuclear Science, Energy and Power Engineering, Shandong University, Jinan 250061, China

**Keywords:** composite phase change materials, thermal safety of battery, skeleton material, nano-morphology regulation

## Abstract

At present, composite phase change materials are widely studied for battery thermal management. However, to ensure the battery’s thermal safety, it is necessary not only to control the temperature during regular operation, but also to prevent sudden thermal runaway. This basic function depends on the flame-retardant properties of the composite phase change materials. In this study, a hexagonal double-layer hollow nanocage S2 with defect orientation was prepared and combined with carbon nanotubes (PNT) derived from polypyrrole (PPy) tubes to form a high adsorption mixture. Multifunctional composite phase change material PNT/S2@PEG/TEP was prepared by adsorbing and coating polyethylene glycol 8000 (PEG-8000) and triethyl phosphate (TEP) with microfibrillated cellulose nanofibers (CNF) as the skeleton. The characterization shows that its thermal conductivity is 0.65 W/m·K and its phase transition enthalpy is 146.1 J/g, demonstrating its excellent thermal regulation. Microcalorimetric testing (MCC) confirmed its flame-retardant ability, attributed to the strong adsorption of PNT/S2 on PEG-8000 and TEP, the improvement in PNT’s thermal conductivity, and the contribution of CNF to flexibility. This composite phase change material, with excellent comprehensive properties, has broad application prospects in thermal safety for electronic equipment, significantly expanding its practical scope.

## 1. Introduction

With the continuous progress of the battery field, especially in core fields such as new energy vehicles, energy storage power stations, and ships, high-energy-density power batteries are emerging one after another. Their outstanding advantages in improving cruising range and expanding energy storage capacity have extensively promoted the green transformation and technological progress in the energy field. At the same time, however, the high-power output characteristics of high-energy density power batteries have brought about the apparent problem of too fast heating rate—the application scenarios of such power batteries are often highly refined. Both the compact battery pack layout of new energy vehicles, the high-intensity working environment of construction machinery, and the closed-space design of ship-borne energy storage equipment feature limited space and insufficient heat-dissipation channels, making it difficult to promptly dissipate the large amount of heat generated during operation. In addition, power batteries often operate under complex conditions, including fast charging, rapid acceleration, low temperatures, and high loads. The superposition of multiple factors makes the battery heat accumulate rapidly, which will not only lead to abnormal temperature rise and affect the key performance of power batteries, such as cycle life, charge, and discharge efficiency, but also lead to thermal runaway, which will lead to thermal abuse accidents, posing a significant threat to equipment safety and personnel safety [1,2]. Therefore, considerable research has been devoted to developing a heat-accumulation mechanism to enhance the energy density of high-energy-density batteries. Currently, there are two mainstream research approaches. One approach is to create an efficient thermal management system; however, the design of such systems often conflicts with the application scenarios that require refinement. Another approach is to develop a composite material with thermal management capabilities. However, this composite material usually has a single function, serving only to adjust the battery temperature without flame-retardant performance, and cannot effectively suppress battery thermal runaway under extreme conditions [3].

As mainstream thermal management materials, composite phase change materials have undergone extensive research and development. However, the current preparation method for these materials typically involves the vacuum impregnation and adsorption of phase change materials into a three-dimensional scaffold. Notably, phase change material encapsulation is achieved solely by leveraging capillary action between the phase change materials and the three-dimensional scaffold’s internal structure [4,5]. Huo et al. [6] prepared the composite phase change material PA/CF, consisting solely of palmitic acid (PA) and copper foam (CF) as the three-dimensional skeleton, by the melt-vacuum impregnation method. Due to the smooth surface of CF, dilute hydrochloric acid can only be used to etch it, increasing its roughness and improving packaging efficiency. Although this method can improve packaging efficiency to some extent, the etching process is often complicated to control precisely, which may significantly degrade the skeleton’s performance. Lu et al. [7] prepared cross-linked veined BN aerogels as a three-dimensional skeleton by the hydrothermal method. They adsorbed PEG via vacuum impregnation to synthesize a composite phase change material, PEG/BN. From the sample photos and synthesis methods of the above two composite phase change materials, PA/CF and PEG/BN, it is evident that they exhibit poor flexibility and occupy substantial space, which do not meet the requirements of refined, high-energy-density battery applications [8,9,10].

Currently, the application scenarios of batteries and battery equipment are becoming increasingly complex. To effectively adjust the working temperature of refined batteries or electronic equipment, and to ensure the thermal safety of batteries or electronic equipment with the ability to withstand severe conditions when thermal failure occurs, the flame retardancy of composite phase change materials with thermal management performance, flame retardancy, and good flexibility has become a research hotspot [11,12,13,14]. The flexibility of composite phase change materials often stems from binders or skeleton-supporting materials, such as polyvinylpyrrolidone (PVP), polyethyleneimine (PEI), and sodium carboxymethyl cellulose (CMC). These materials can bring some flexibility and shape stability to composite phase change materials, but their flexibility performance is often limited. Additionally, various other materials and processes are employed to prepare flexible composite phase change materials. Yang et al. [15] formed a special cross-linking agent by the polymerization reaction between chitosan quaternary ammonium salt (CQS) and PEI, and used MXene nanosheets as heat conducting materials, and then combined them by a quantitative freezing process to prepare a “fence-like” three-dimensional block supporting material. Due to the presence of a cross-linking agent, this type of skeleton material exhibits some flexibility, which is significantly reduced after absorbing phase change materials. Sun et al. [16] added a mixture of liquid phase change material PEG-400 and solid phase change material PEG-2000 into sodium stearate (NaR) nanoparticles to prepare a gel composite phase change material. The composite phase change material prepared by this process exhibits some flexibility. However, the phase change enthalpy of PEG is directly proportional to its molecular weight. The presence of PEG-400 in this composite material reduces its phase-change enthalpy, limiting its temperature adjustment range. Hu et al. [17] utilized a continuous heat-conduction network structure formed by one-dimensional carbon fiber, combined with SBS thermoplastic elastomer, to produce a skeleton material with high flexibility and thermal conductivity. However, the adsorption capacity of this skeleton material is limited, and the amount of phase change materials that can be adsorbed is limited. Although the composite phase change materials prepared by the aforementioned method exhibit favorable thermal conductivity and flexibility, their phase change enthalpy remains relatively low. Consequently, there is an urgent need to explore appropriate materials and optimized fabrication processes to develop composite phase change materials that meet the demands of complex application scenarios [18,19,20].

In this study, nanocage S2 with a regular hexagonal, double-layer hollow structure was prepared through fine control of its microstructure, resulting in a high adsorption capacity. Combined with special carbon nanotube PNT and skeleton reinforcing agent CNF, which also possess strong adsorption capacity and high thermal conductivity, they were used to adsorb PEG-8000 and TEP. Finally, a high-performance composite phase change material, PNT/S2@PEG/TEP, with both thermal and flame-retardant capabilities, was prepared. After numerous tests, it has been shown that the PNT/S2 mixture exhibits a strong adsorption capacity. PNT/S2@PEG/TEP is a homogeneous composite phase change material, and its high enthalpy of phase change and high thermal conductivity enable it to exhibit excellent thermal management capabilities. The MCC test demonstrates its flame-retardant performance, and the flexibility provided by CNF makes it applicable to various thermal-safety scenarios for electronic equipment.

## 2. Materials and Methods

### 2.1. Materials

Pyrrole (AR); FeCl_3_·6H_2_O (AR); Methyl orange (AR); Tetrapropyl ammonium hydroxide aqueous solution (TPAOH) (1.0 M in water); Ethyl orthosilicate (98 wt%); Ammonia water (AR); PEG-8000 (Mn = 8000 g/mol, 99 wt%) CNF (3 wt% in water).

### 2.2. Preparation of PNT and S2

The PPy tube was oxidized with FeCl_3_ using the classical one-pot oxidation method, and the pH of the mixed solution was stabilized with methyl orange, allowing PPy to be prepared. A special carbon nanotube PNT [21] containing nitrogen was prepared by pyrolysis of PPy in a nitrogen environment.

10 mL of TPAOH was mixed with 50 mL of H_2_O, and then 10 mL of tetraethyl orthosilicate was added to the mixture. The mixture was stirred for 4 h. The obtained solution was transferred to a 100 mL high-temperature and high-pressure reactive still and crystallized at 170 °C for 24 h. After several centrifugal washes and drying, S-1 crystals were obtained. 0.6 g of the S-1 crystal thus obtained, 2.5 mL of TPAOH, and 2.5 mL of ethyl orthosilicate were dispersed in 50 mL of H_2_O. The mixture was stirred for 4 h. The obtained solution was transferred to a 100 mL reactive still and crystallized at 170 °C for 24 h. After several centrifugal washes and drying, S-1@S-1 crystals were obtained. Then the suspension was transferred to a 100 mL reactive still and crystallized at 160 °C for 24 h. The product was separated by centrifugation, washed with H_2_O and ethanol several times, dried in an oven at 60 °C overnight, and then calcined at 550 °C for 6 h to obtain the final product S2 with a double-layered hexagonal hollow structure.

### 2.3. Preparation of PNT/S2@PEG/TEP and PNT@PEG/TEP

**Figure 1:**
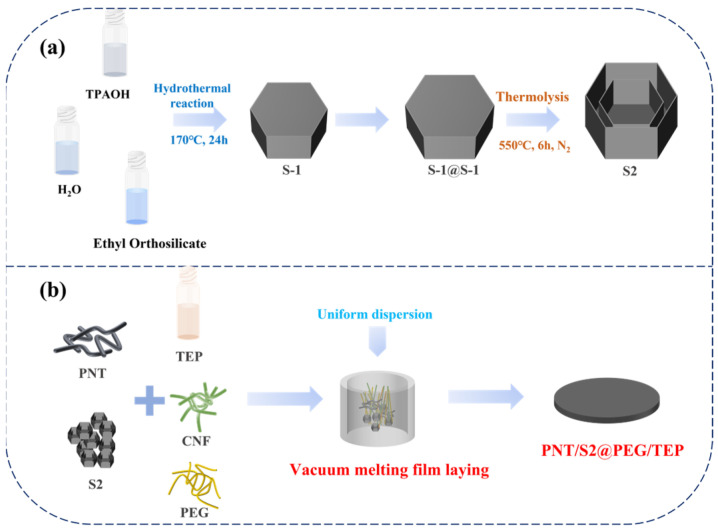
(**a**) Schematic diagram of preparation process of S2; (**b**) Schematic diagram of preparation process of PNT/S2@PEG/TEP.

1 g of PNT powder was mixed with 0.5 g of S2 powder in 200 mL of H_2_O and ultrasonically treated to form a uniform suspension, then 20 g of PEG-8000 and 10 mL of TEP were added to the suspension, stirred for 30 min, then 30 g of CNF was added, stirred at 1000 rpm for 2 h, and then the mixed suspension was poured into a mold. Then, the mold was placed in a vacuum oven at 80 °C for 24 h to undergo vacuum drying. Next, the obtained product was heated in a blast oven at 80 °C for 2 h to remove PEG-8000 and TEP that could not be firmly adsorbed, thereby obtaining PNT/S2@PEG/TEP. The schematic diagram of the preparation process is shown in Figure 1. A total of 0.5 g of S2 powder was replaced by 0.5 g of PNT powder, and PNT@PEG/TEP could be obtained under the same conditions. 1 g of PNT powder was replaced with 1 g of PEG under the same conditions, and PEG/TEP was obtained.

### 2.4. Characterization Testing Technology and Related Instruments

See Supplementary Information for test and characterization methods and technical details.

## 3. Results

### 3.1. Microstructure Characterization of PNT/S2@PEG/TEP and PNT@PEG/TEP Composites Films

**Figure 2:**
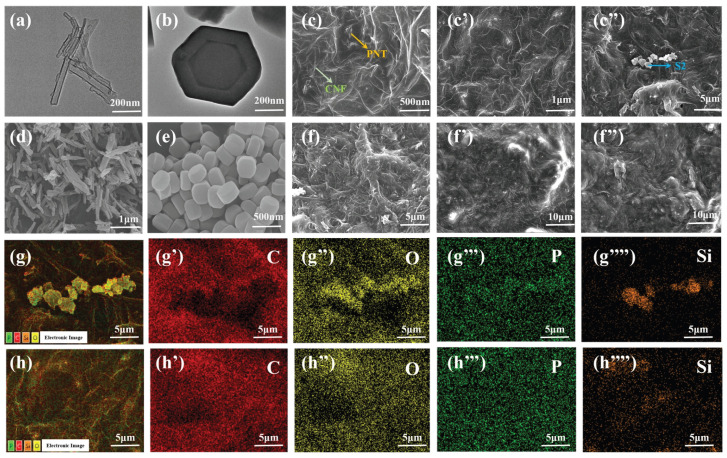
(**a**) TEM images of PNT; (**b**) TEM images of S2; (**c**–**c’’**) SEM images of PNT/S2@PEG/TEP; (**d**) SEM image of the PNT; (**e**) SEM image of the S2; (**f**–**f’’**) SEM image of the PNT@PEG/TEP; (**g**–**g’’’’**) Local surface SEM image of PNT/S2@PEG/TEP, along with the EDS elemental mapping of C, O, P, and Si; (**h**–**h’’’’**) Local surface SEM image of PNT@PEG/TEP, along with the EDS elemental mapping of C, O, P, and Si.

The microstructure of PNT/S2@PEG/TEP and PNT@PEG/TEP composites is shown in Figure 2. From the TEM images of PNT and S2 in Figure 2a,b, PNT has an obvious tubular structure, and the regular hexagonal double-layer hollow structure of S2 is also clearly visible. The tubular structure of PNT and the regular hexagonal structure of S2 can also be clearly observed in Figure 2d,e. The existence of PNT and CNF can be observed in Figure 2c–c’’ and Figure 2f–f’’, and the existence of S2 can be clearly observed in Figure 2c’’. After detecting PNT/S2@PEG/TEP and PNT@PEG/TEP by EDS, the existence of the P element can be clearly seen, which comes from the uniform distribution of TEP on the two composites. The Si content difference can further support the S2 content difference between them.

**Figure 3:**
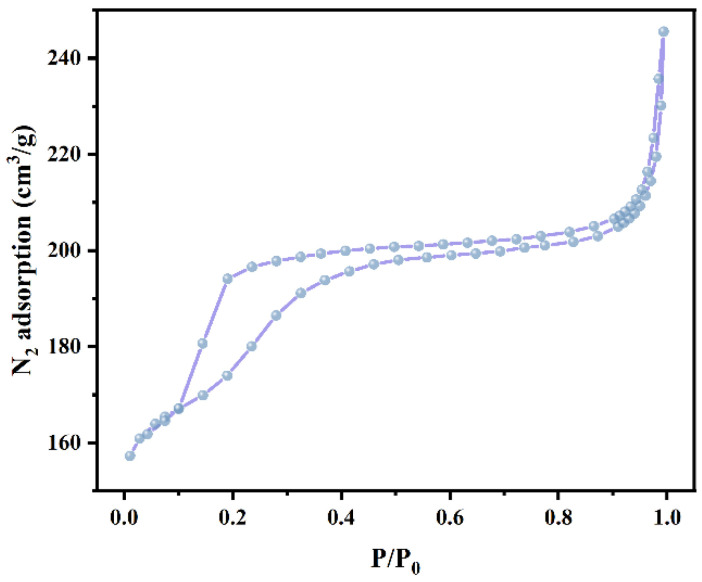
N_2_ absorption curve of PNT/S2.

The N_2_ adsorption–desorption curve of PNT/S2 (the mass ratio of PNT to S2 is 2:1) is shown in Figure 3. According to the BET test results, the specific surface area of PNT/S2 reaches 672.6307 m^2^/g, consistent with the tubular structure and the regular hexagonal double-layer hollow structure observed in the TEM images of PNT and S2.

### 3.2. Structure Analysis of PNT/S2@PEG/TEP and PNT@PEG/TEP Composites Films

**Figure 4:**
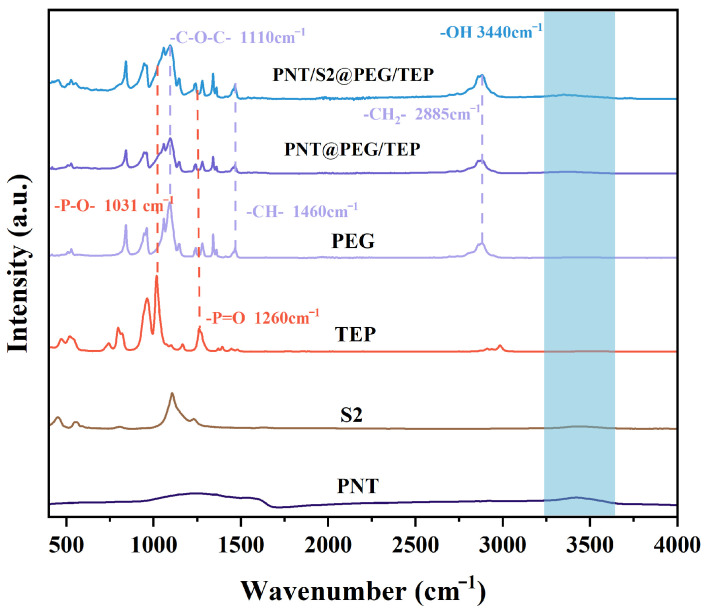
The FT-IR spectra of PNT, S2, TEP, PEG, PNT@PEG/TEP, and PNT/S2@PEG/TEP, respectively.

Fourier transform infrared spectroscopy (FT-IR) was employed to characterize PNT, S2, TEP, PEG, PNT@PEG/TEP, and PNT/S2@PEG/TEP, with the corresponding results presented in Figure 4. Notably, all samples exhibit a characteristic peak at 3380 cm^−1^, which is attributed to the O-H vibration [22]. For PEG, its characteristic absorption peaks at 2878 cm^−1^, 1460 cm^−1^, 1340 cm^−1^, and 1100 cm^−1^ are associated with the stretching vibrations of -CH_2_, C-H, and C-O-C groups [23]. Additionally, prominent peaks observed at approximately 1260 cm^−1^ and 1031 cm^−1^ correspond to the -P=O and -P-O- groups, respectively, providing further evidence for the presence of TEP in the composite materials [24].

**Figure 5:**
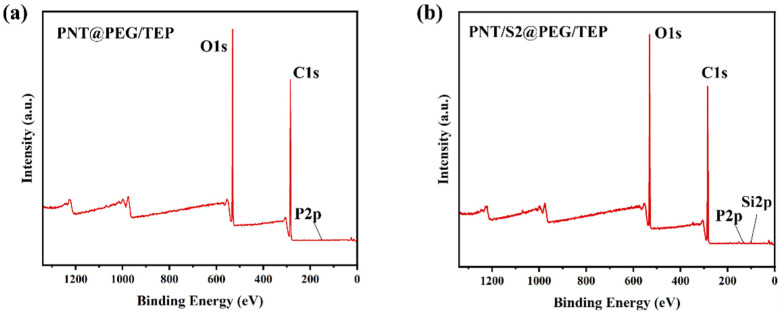
XPS survey spectra of PNT@PEG/TEP (**a**) and PNT/S2@PEG/TEP (**b**).

The difference in surface chemical composition between PNT@PEG/TEP and PNT/S2@PEG/TEP composites was identified by XPS, as shown in Figure 5. Figure 5a,b are XPS broad-spectrum diagrams of PNT@PEG/TEP and PNT/S2@PEG/TEP composite films, respectively. In the measurement area (0–1300 eV), typical peaks of C 1s, O 1s, and P 2p were observed. The results showed that TEP was adequately mixed into PEG by the melt coating process. The PNT/S2@PEG/TEP sample exhibited an additional Si 2p peak; however, its intensity was low due to its limited content. The existence of the Si 2p peak indicated that S2 was successfully complexed into PEG.

### 3.3. Thermal Management Performance of Composite Films

**Figure 6:**
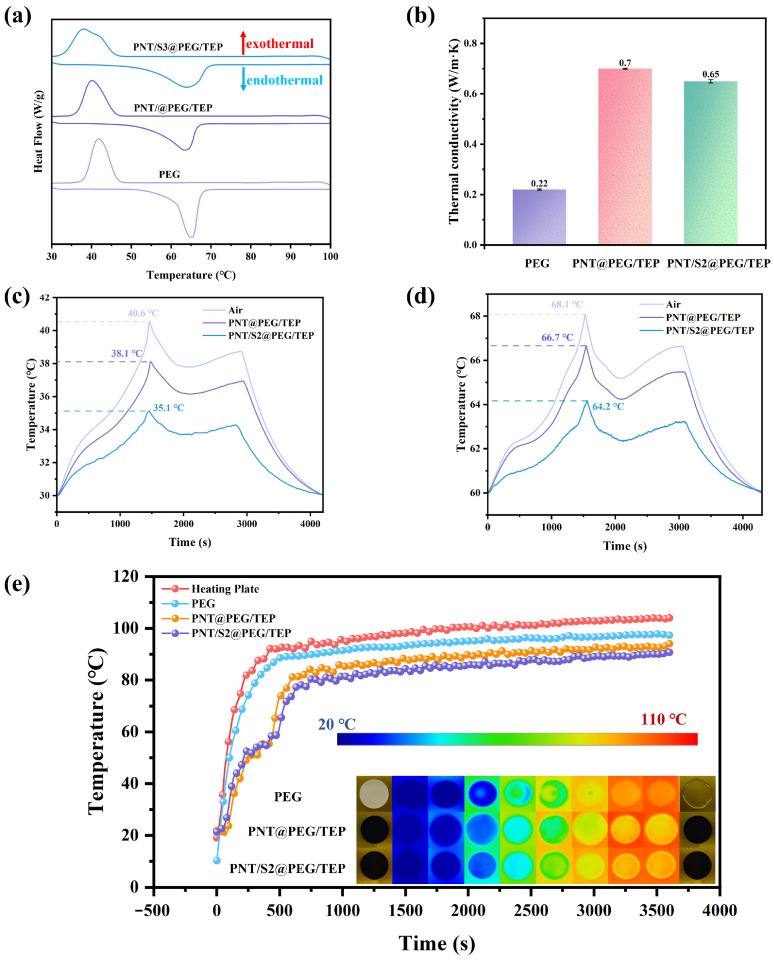
(**a**) The DSC curves of PEG, PNT/S2@PEG/TEP, and PNT@PEG/TEP, respectively; (**b**) Thermal conductivity of PEG, PNT/S2@PEG/TEP, and PNT@PEG/TEP; (**c**,**d**) Surface temperature of battery at 30 °C and 60 °C; (**e**) Time–temperature curve of the heating plate, PEG, PNT/S2@PEG/TEP, and PNT@PEG/TEP at different times.

Figure 6a presents the DSC curves of PEG and its corresponding composites, while Table 1 summarizes the melting point (T_m_) and enthalpy (ΔH_m_) data derived from the DSC tests. PEG exhibits prominent endothermic behavior, with a Tm of 62.7 °C and ΔH_m_ of 180.4 J/g, confirming its suitability as a high-performance energy storage and endothermic material. In contrast, the composites show a slight reduction in phase change enthalpy, attributed to the incorporation of functional components (PNT, S2, TEP, and CNF), none of which undergo phase transitions within the 30–100 °C range. Additionally, the composites differ from pure PEG in phase transition behavior, displaying a lower T_m_. This phenomenon demonstrates that the composite’s special structure can regulate the free movement of molten PEG during the phase transition, and this regulatory effect is enhanced by the synergistic effects of PNT, S2, and CNF, as well as by the composite’s unique structure. The difference in phase transition enthalpy between PNT/S2@PEG/TEP and PNT@PEG/TEP may be attributed to the variation in S2 content, which also demonstrates S2’s strong adsorption capacity.

Thermal conductivity is a critical indicator of the heat transfer efficiency of composite phase change materials. In this study, the thermal conductivity of PEG and its composites was characterized, and the results are presented in Figure 6b. The measured thermal conductivities of the three materials are 0.22 W/m·K, 0.7 W/m·K, and 0.65 W/m·K, respectively. This enhancement (relative to pure PEG) stems from the uniform dispersion of the PNT/S2 network within the PEG matrix.

Through the battery thermal management system, the ability of composite materials to regulate battery temperature and conduct effective battery thermal management was tested. The tests were conducted in constant temperature and humidity boxes at 30 °C and 60 °C, respectively. The two composite materials were wrapped around 18650 lithium batteries with the same model and SOC of 100%, and thermocouples were attached to the battery surface. The control group was an unwrapped battery. The battery was charged and discharged at a rate of 2C. The specific test parameters are shown in Table S2, and the test system is shown in Figure S1. The test results are shown in Figure 6c,d. Obviously, both composites possess thermal management capabilities, but the PNT/S2@PEG/TEP composite exhibits superior thermal management at two different working temperatures. The reason for this phenomenon may be that although the thermal conductivity of PNT/S2@PEG/TEP is slightly lower than that of PNT@PEG/TEP, the more complex microstructure of PNT/S2@PEG/TEP provides more channels for heat transfer, which enables the overall composite to achieve faster and more uniform thermal response, thus exerting better thermal management performance [25,26,27,28]. PNT/S2@PEG/TEP can be cut into the required shape and maintain its shape stability. Moreover, PNT/S2@PEG/TEP with different sizes can be prepared by adjusting the die size. The PNT/S2@PEG/TEP prepared this time can be perfectly wrapped and attached to the surface of an 18650 battery, meeting the application requirements for battery thermal management. The related image is shown in Figure S1. In addition, the time–temperature curve and DSC curve of the two composites after 30 battery heating/cooling cycles are shown in Figure S4, and the DSC data are shown in Table S2.

By analyzing the temperature–time curve and infrared image of the sample during heating, the composite film’s temperature control performance under direct heating can be observed intuitively (see Figure 6e). During testing, the samples were placed on a metal heating plate, and an infrared thermal imager was used to record the temperature distribution on the surfaces of the PEG, PNT@PEG/TEP and PNT/S2@PEG/TEP composite films over 3600 s. At the initial stage of the test, the temperature of all composite films was 20 °C. Subsequently, the heating plate was gradually raised to 105 °C. The color of the composite films changed noticeably, indicating that their surface temperature increased continuously over time. It is worth noting that PEG begins to melt at a specific temperature and eventually reaches a molten state.

The temperature change curve and image change show that the surface temperatures of PEG, PNT@PEG/TEP, and PNT/S2@PEG/TEP are consistently lower than the heating plate temperature for the first 3600 s, and the two samples differ only slightly. The surface temperatures of PNT/S2@PEG/TEP are always 2–3 °C lower than those of PNT@PEG/TEP. Although the surface temperature of pure PEG has always been lower than that of the heating plate, its poor thermal conductivity leads to an uneven surface temperature distribution on the heating plate during rapid heating. It has completely melted on the heating plate after 3600 s of heating. Its shape stability is poor, whereas PNT@PEG/TEP and PNT/S2@PEG/TEP exhibit essentially no morphological changes before and after heating. When the surface temperature of the PNT@PEG/TEP and PNT/S2@PEG/TEP reached the phase transition temperature, an apparent temperature plateau appeared in the temperature curve, indicating that both samples exhibited good thermal response speed [29,30,31]. The infrared thermal imaging results for the two samples also show that they exhibit excellent thermal management performance. The surface temperature curves of the samples and the heating plate have been smoothed properly.

**Figure 7:**
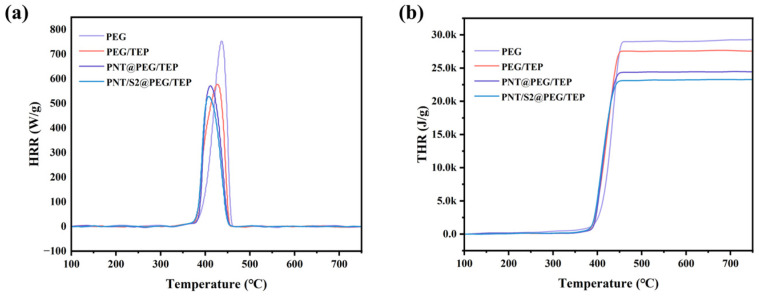
(**a**) The HRR curves of PEG, PEG/TEP and PNT/S2@PEG/TEP, respectively; (**b**) The THR curves of PEG, PEG/TEP, PNT@PEG/TEP and PNT/S2@PEG/TEP, respectively.

The flame-retardant properties of PEG/TEP, PNT/S2@PEG/TEP and PNT@PEG/TEP were quantitatively tested by MCC. The results of MCC are shown in Table 2 and Figure 7. It can be clearly seen that the heat release (HR), heat release rate (HRR), and total heat release (THR) of PNT/S2@PEG/TEP are significantly lower than those of PEG, which is due to the addition of flame-retardant TEP, which makes the composites have a specific flame-retardant ability. The HRR and HR of PEG/TEP are obviously much lower than those of pure PEG, and CNF, as a macromolecular polysaccharide material, is inevitably flammable, so the reason for this phenomenon can be basically determined because TEP plays an inhibitory role in the rapid pyrolysis of the composite material. The flame-retardant properties of PNT/S2@PEG/TEP and PNT@PEG/TEP make them potentially suitable for use in harsher thermal environments [32,33,34,35].

## 4. Conclusions

Focusing on the core requirements in the field of thermal safety of high-energy density batteries, this study successfully developed PNT/S2@PEG/TEP composite phase change materials with high thermal management and reliable flame retardancy, aiming at the problems of single function (only temperature adjustment but no flame retardancy), insufficient flexibility, and weak ability to adapt to refined application scenarios.

The nanocage S2, with a regular hexagonal double-layer hollow structure, was prepared by precisely controlling the microstructure using a defect-oriented method. At the same time, PPy was prepared by a classical one-pot oxidation method and pyrolyzed in nitrogen. A special carbon nanotube PNT with high adsorption capacity and high thermal conductivity was obtained. Using CNF as a skeleton reinforcing agent, PNT and S2 were mixed in a predetermined ratio to form an adsorption carrier, and PEG-8000 and TEP were adsorbed and encapsulated via vacuum drying and other processes, finally creating a multifunctional composite phase change material. The structural characterization confirmed that the specific surface area of the PNT/S2 mixture was as high as 672.6307 m^2^/g, and the tubular and hollow structures endowed it with strong adsorption capacity. EDS, FT-IR, and XPS tests showed that all components were physically combined evenly, and TEP and S2 were successfully integrated into the system. The performance test shows that the composite’s thermal conductivity reaches 0.65 W/m·K, which is twice that of pure PEG, and its enthalpy of phase change is 146.1 J/g, indicating a rapid and uniform thermal response. The microcalorimetric test revealed that its heat release rate and total heat release were significantly lower than those of pure PEG, indicating an excellent flame-retardant effect. In addition, CNF endows the material with good flexibility and shape stability, and it can be cut to fit closely to the surface of an 18650 battery. In the 2C rate charge–discharge test at 30 °C and 60 °C, the thermal management performance is better than that of the control sample without S2.

This material innovatively achieves the synergy of thermal management and flame-retardant functionalities, addressing the limitations of traditional thermal management materials (single functionality and poor adaptability). Endowed with flexibility and adaptability, it effectively expands the application scenarios of composite phase change materials for thermal safety in various electronic devices. Furthermore, it provides a novel solution for the thermal safety protection of high-energy-density batteries and precision electronic equipment, boasting substantial application potential.

## Figures and Tables

**Table 1:** Phase change properties of PEG and composite films.

Sample	Heating Process		Cooling Process	
	H_m_ (J/g)	T_m_ (°C)	H_c_ (J/g)	T_c_ (°C)
PEG	180.4	61.2	166.5	46.3
PNT@PEG/TEP	139.9	56.2	141.3	45.8
PNT/S2@PEG/TEP	146.1	55.5	149.7	45.7

**Table 2:** MCC data for PEG and composite films.

Sample	HR (J/g·K)	HRR (W/g)	THR (kJ/g)	Temperature (°C)
PEG	745.7	753.2	26.1	436.6
PEG/TEP	572.0	577.8	24.2	428.1
PNT@PEG/TEP	565.9	571.5	22.8	412.2
PNT/S2@PEG/TEP	522.8	528.0	23.0	408.3

## Data Availability

Data will be made available on request.

## Supplementary Materials

The following supporting information can be downloaded at: https://www.mdpi.com/article/10.3390/nano16010026/s1, Figure S1. Schematic diagram of battery charging and discharging temperature test device; Figure S2. (a) Sample photos of PNT/S2@PEG/TEP and photos of samples cut by scissors; (b) Flexibility and thickness of PNT/S2@PEG/TEP; Table S1. Parameters setting of battery charge and discharge processing; Figure S3. P 2pXPS narrow scanning spectrograms of PNT@PEG/TEP (a) and PNT/S2@PEG/TEP (b); Figure S4. (a) Temperature curves of PNT @ PEG/TEP and PNT/S2@PEG/TEP after 30 heating and cooling cycles; (b) DSC curves of PNT @ PEG/TEP and PNT/S2@PEG/TEP after 30 heating and cooling cycles; Figure S5. Morphology of PNT/S2@PEG/TEP after 30 heating and cooling cycles; Table S2. Phase transformation performance of PNT@PEG/TEP and PNT/S2@PEG/TEP after 30 heating and cooling cycles; Table S3. Mass changes in PEG, PNT@PEG/TEP and PNT/S2@PEG/TEP; Figure S6. Morphological changes in PEG, PNT@PEG/TEP and PNT/S2@PEG/TEP.

## Abbreviations

The following abbreviations are used in this manuscript:

PPy	Polypyrrole
PEG-8000	Polyethylene glycol 8000
TEP	Triethyl phosphate
CNF	Microfibrillated cellulose nanofibers
PA	Palmitic acid
CF	Copper foam
PVP	Polyvinylpyrrolidone
PEI	Polyethyleneimine
CMC	Sodium carboxymethyl cellulose
CQS	Chitosan quaternary ammonium salt
TPAOH	Tetrapropyl ammonium hydroxide aqueous solution
TEM	Transmission Electron Microscope
SEM	Scanning electron microscope
EDS	Energy dispersive spectroscopy
FT-IR	Fourier transform infrared spectroscopy
DSC	Differential Scanning Calorimetry
SOC	State of Charge
MCC	Microcalorimeter
HR	Heat release
HRR	Heat release rate
THR	Total heat release
MCC	Micro calorimeter
