# Supplementary material for: Construction of Regular Hexagonal Double-Layer Hollow Nanocages by Defect Orientation and Composite Phase Change Materials with Carbon Nanotubes for Thermal Safety of Power Batteries
Source: Nanomaterials (Basel). 2025 Dec 24;16(1):26. doi: 10.3390/nano16010026 (PMC12787748; doi:10.3390/nano16010026)
Supplement: Supplementary file 1 [file nanomaterials-16-00026-s001.zip › nanomaterials-4033602-supplementary.pdf]

nanomaterials

MDPI

Supplementary Material

Construction of regular hexagonal double-layer hollow nanocages by defect orientation and composite phase change materials with carbon nanotubes for thermal safety of power batteries

Silong Wang [1], Wei Yan [1,*], Pan Sun [1] and Jun Yan [1]

[1] 1 School of Nuclear Science, Energy and Power Engineering, Shandong University, Jinan 250061, Shandong, China.

* Correspondence: Corresponding Author: yanwei@sdu.edu.cn (Wei Yan);

Characterization testing technology and related instruments

The microstructures of the samples were obtained by SEM (JEOL JSM-7001F). A field emission SEM also obtained the energy dispersive spectroscopy (EDS) elemental mappings of samples.

The specific surface area of PNT/S2 was measured by American Micromeritics ASAP 2460 automatic specific surface and porosity analyzer, and its pore size distribution and specific surface area were analyzed.

The chemical structures of the PNT, S2, TEP, PEG, PNT@PEG/TEP and PNT/S2@PEG/TEP were analyzed by KBr sampling, Fourier transform infrared spectroscopy (FT-IR, whose type was Bruker Vertex 70).

The element composition and chemical bond of composite phase change materials were analyzed by Thermo Scientific K-Alpha X-ray electron spectrometer in the United States and Mono AlKα source (Al Kα source).

The latent heat and temperature of phase transition were obtained by analyzing results measured from DSC (calorimeter model: METTLER TOLEDO DSC2). All the samples were tested in a nitrogen atmosphere with a flow rate of 20 mL/min, and in the temperature range of 20-100°C with a heating rate of 10 °C/min.

The thermal conductivity of the samples was measured using a thermal conductivity meter (whose type was TC3000E, XIATECH, China). At least four measurements were taken for each sample to ensure the repeatability of the results.

The infrared images of the composite membranes were obtained by Fotric 326+ infrared thermal imager (FOTRIC – Shanghai Thermal Image Science & Technology Company, China).

The combustion characteristics of the samples were measured by a microcalorimeter, and the heat of combustion and combustion rate released by the sample were measured at a heating speed of 1°C/min at 30-700°C.

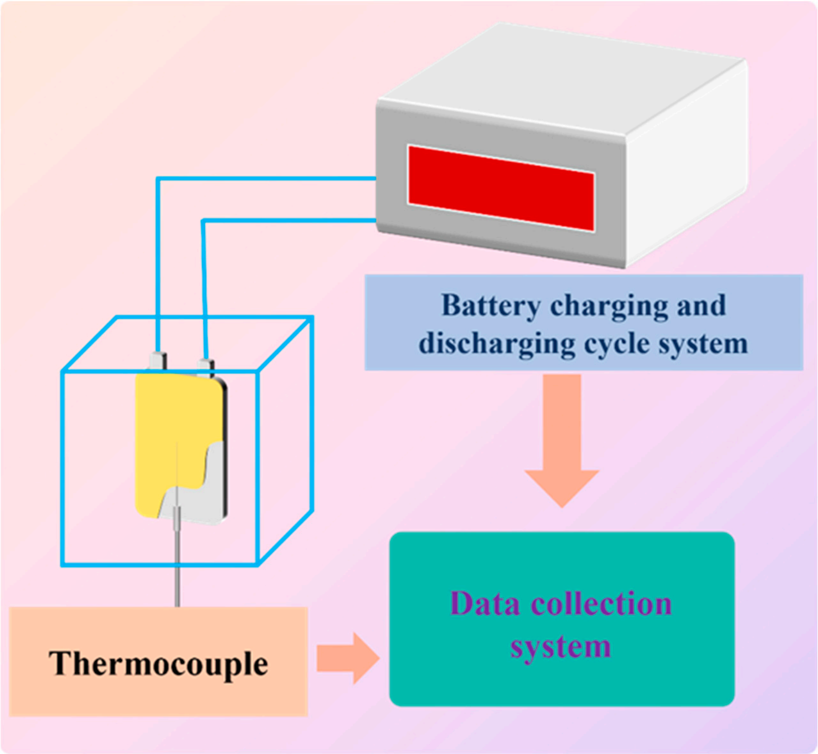

Figure S1. Schematic diagram of battery charging and discharging temperature test device.

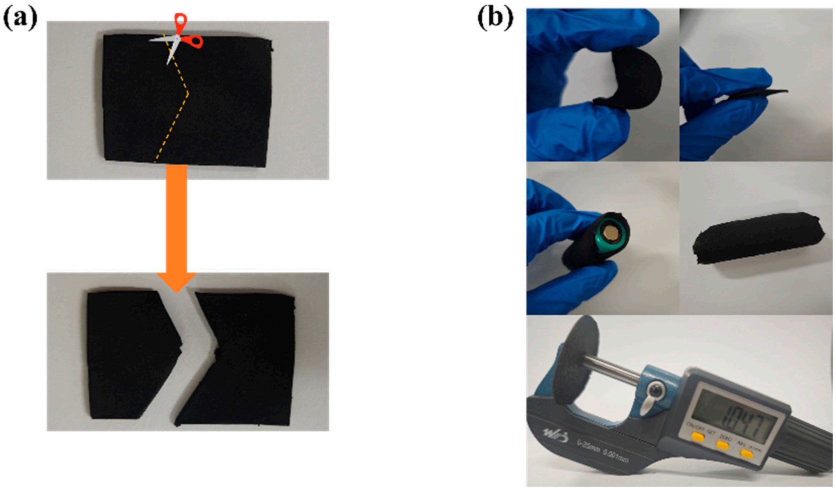

Figure S2. (a) Sample photos of PNT/S2@PEG/TEP and photos of samples cut by scissors; (b) Flexibility and thickness of PNT/S2@PEG/TEP.

Table S1. Parameters setting of battery charge and discharge processing.

Parameters / Procedures	Voltage (V)	Current (mA)
Constant current charging	4.2	4400
Constant voltage charging	4.2	100 (cutoff current)
Constant current discharging	4.2	4400
Standing	20 minutes	

https://doi.org/10.3390/nano16010026

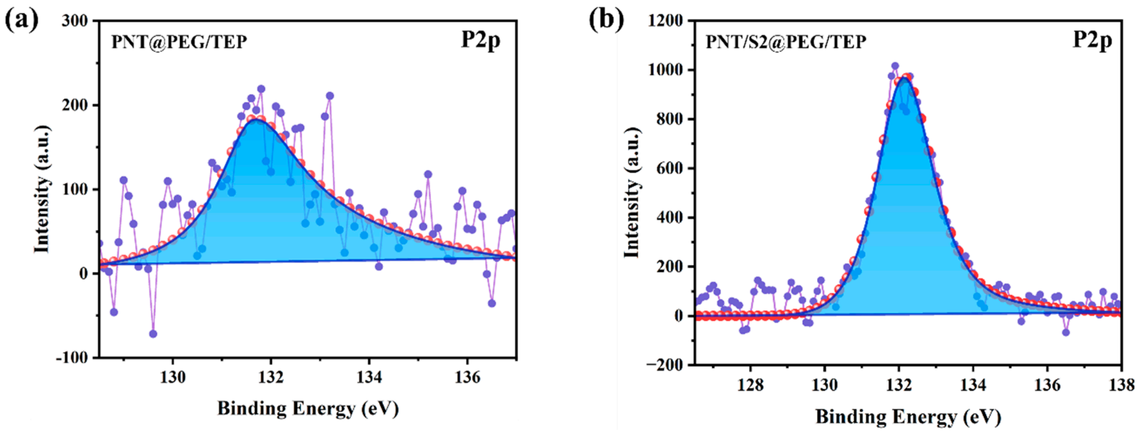

Figure S3. P 2pXPS narrow scanning spectrograms of PNT@PEG/TEP (a) and PNT/S2@PEG/TEP (b).

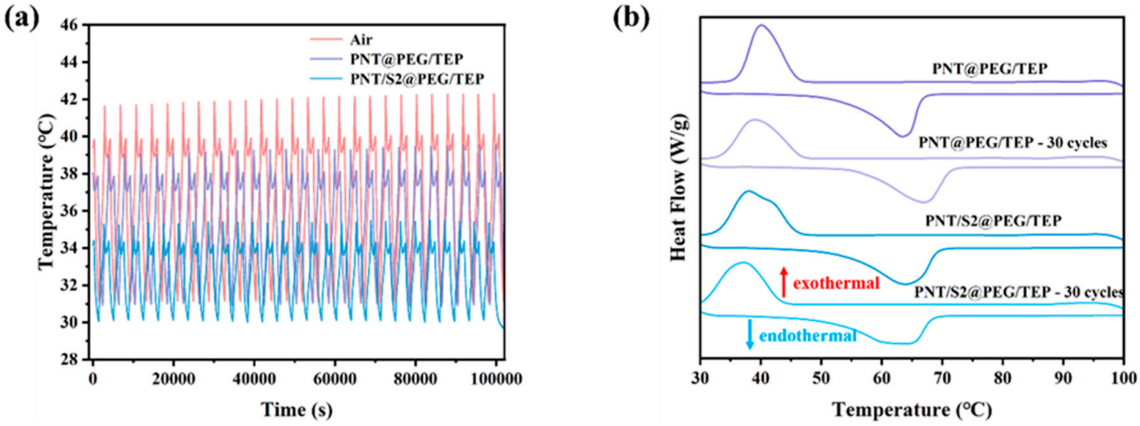

Figure S4. (a) Temperature curves of PNT @ PEG/TEP and PNT/S2@PEG/TEP after 30 heating and cooling cycles; (b) DSC curves of PNT @ PEG/TEP and PNT/S2@PEG/TEP after 30 heating and cooling cycles.

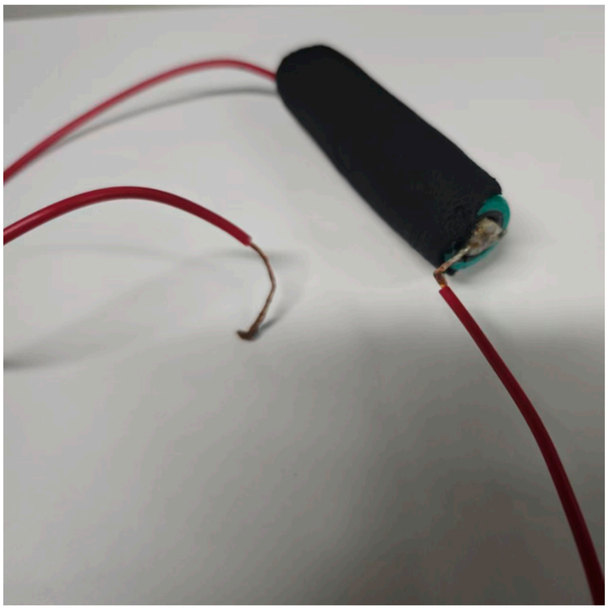

Figure S5. Morphology of PNT/S2@PEG/TEP after 30 heating and cooling cycles.

Table S2. Phase transformation performance of PNT@PEG/TEP and PNT/S2@PEG/TEP after 30 heating and cooling cycles.

https://doi.org/10.3390/nano16010026

Sample	Heading process		Cooling process	
	H_m (J/g)	T_m (°C)	H_c (J/g)	T_c (°C)
PNT@PEG/TEP - 30 cycles	130.4	57.1	130	46.7
PNT/S2@PEG/TEP - 30 cycles	143.4	52.7	131.9	43.2

Table S3. Mass changes in PEG, PNT@PEG/TEP and PNT/S2@PEG/TEP.

Samples	Mass at Room temperature	Mass at 80°C
PEG	2.55g	0g
PNT@PEG/TEP	2.07g	2.01g
PNT/S2@PEG/TEP	2.5g	2.49g

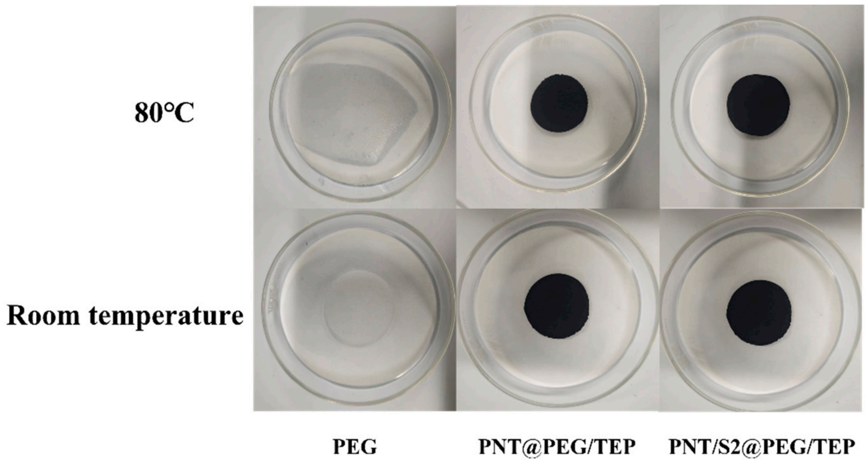

Figure S6. Morphological changes in PEG, PNT@PEG/TEP and PNT/S2@PEG/TEP.

https://doi.org/10.3390/nano16010026
